# Excitation-Power-Dependent Color Tuning in a Single Sn-Doped CdS Nanowire

**DOI:** 10.3390/molecules29225389

**Published:** 2024-11-15

**Authors:** Ye Tian, Shangfei Yao, Bingsuo Zou

**Affiliations:** 1 School of Semiconductor and Physics, North University of China, Taiyuan 030051, China; 2 State Key Laboratory of Featured Metal Materials and Life-Cycle Safety for Composite Structures, School of Resources, Environments and Materials, Guangxi University, Nanning 530004, China; yaoshangfei@st.gxu.edu.cn

**Keywords:** Sn-doped CdS nanowire, trap-state emission, microcavity, excitation power, color tuning

## Abstract

Multicolor emission and dynamic color tuning with large spectral range are challenging to realize but critically important in many areas of technology and daily life, such as general lighting, display, multicolor detection and multi-band communication. Herein, we report an excitation-power-dependent color-tuning emission from an individual Sn-doped CdS nanowire with a large spectral range and continuous color tuning. Its photoluminescence (PL) spectrum shows a broad trap-state emission band out of Sn dopants, which is superposed by whispering-gallery (WG) microcavity due to the nanostructure size and its structure, besides the CdS band-edge emission. By simply changing the excitation power from 0.25 to 1.36 mW, we demonstrate that the typical Sn-doped CdS nanowire with the weight ratio of 10:1 of CdS and SnO_2_, the emission color can change from red to orange to yellow to green. In view of the stable properties and large spectral range, the Sn-doped CdS nanowires are very promising potential candidates in nanoscale optoelectronic devices.

## 1. Introduction

The one-dimensional (1D) CdS nanostructures are excellent materials for optoelectronic applications owing to their advantages, such as large specific surface area [1], natural microcavity structure, significant quantum size effect [2] and excellent light-emitting characteristics [3]. The applications of the 1D CdS nanostructures include lasers [4,5], waveguide devices [6,7], Light-Emitting Diodes (LEDs) [8,9] and rich-color displays [10,11]. Recently, excitation-power-dependent color tuning from transition metal ion-doped CdS nanostructures has attracted much attention. The color tuning is usually determined by the mechanism for the saturation of transition metal ion-dopant emission [12]. For transition metal ion-dopant CdS nanostructures, a portion of photo-generated excitons in CdS can transferred to transition metal ion dopants, leading to a down-conversion emission at transition metal ion through their trap-state emission or d-d transition. Transition metal ion-doped CdS nanostructures can thus exhibit dual color emissions, including high-energy band-edge emission and low-energy trap-state emission. Spectroscopic studies have shown that the PL lifetime of transition metal ion dopant (τ dopant) is much longer than that of CdS band-edge excitons (τ excitons), and the lifetime between transition metal ion dopant and band-edge excitons is greatly unbalanced [13]. This imbalance provides a way to tune the emission color of transition metal ion-doped CdS nanostructures by simply changing the excitation power. Such excitation-dependent color-tuning emission has been observed in Mn^2+^, Zn^2+^, In^+^ and Sn^2+^ doped CdS nanostructures [14,15]. In the study of excitation-power-dependent color tuning, one of the focuses of the researchers is on the spectral range [16].

The size of the spectral range of excitation-power-dependent color tuning is usually expressed by the area of the color composition range area in the Commission International de l’Eclairage (CIE) chromaticity diagram [17]. The spectral range of color tuning is determined by the center wavelength and the number of light-emitting peaks. For Mn^2+^, Zn^2+^ and In^+^ doped CdS nanostructures, they all have two light-emitting peaks, and their light-emitting peaks are centered at 530 nm and 620 nm, respectively. The spectral range of these nanostructures is a line in the CIE chromaticity diagram. For example, due to the limitation of the color spectrum, Mn-doped CdS can reflect red and green lighting with the increase of excitation power, but the intermediate colors of orange and yellow are difficult to display. Recently, research has shown that the combination of four separate lasers with different wavelengths, compared to the combination of two separate lasers with different typical wavelengths, can increase the area of the color composition range area in the CIE chromaticity diagram, thus increasing the spectral range [18,19,20]. As a result, increasing the number of light-emitting peaks is an effective way to widen the spectral range of excitation-power-dependent color tuning. For the Sn-doped CdS nanowires, lighting emission of different wavelengths in the trap state has been observed due to the microcavity effect [21,22,23,24], which may lead to an increase in the number of light-emitting peaks. Meanwhile, Song, G.L. et al. found the color tuning process from red to green in the Sn-doped CdS multi-branched nanostructure. However, this multi-branched nanostructure has no obvious microcavity effect [25,26], and the intermediate colors of orange and yellow are difficult to display. Therefore, using Sn-doped CdS nanowires may be a solution to enhance the spectral range of excitation-power-dependent tuning, and the intermediate colors in the color-tuning process may be more easily displayed.

The emission color of the color-tuning process in transition metal ion-doped CdS nanostructures is determined by the ratio of the emission intensity between the band-edge emission (I_band-edge_) and the trap-state emission (I_trap-state_) [27,28]. As the excitation power changes, a change in the the ratio of Iband-edge and the I trap-state emission can be obtained, so the emission color will change. When the excitation power is low, due to the effective energy transfer from excitons to the dopant process, their emission color is mainly controlled by trap-state emission (I_trap-state_ >> I_band-edge_). When the excitation power increases to a certain extent, there is a process of I band-edge ≈ I trap-state. At this time, their emission color is mixed together by band-edge emission and trap-state emission. When the excitation power is large enough, their emission color is dominated by the band-edge emission (I_band-edge_ >> I_trap-state_). However, not all transition metal ion-doped CdS nanostructures can achieve color tuning with the change of excitation power. When the transition metal ion-doped CdS nanostructures have a low transition metal ion-doped concentration, the intensity of the trap state is almost negligible. The ratio of the emission intensity band-edge emission and trap-state emission has always tended to infinity, and the color emission is always green with the increase of excitation power. When the transition metal ion-doped CdS nanostructures with over-doping concentration, the intensity of band-edge emission is almost negligible. The ratio of the emission intensity band-edge emission and trap-state emission has always tended to zero, and the color emission is always red with the increase of excitation power. Therefore, controlling the transition metal ion doping concentration in CdS nanostructures plays an important role in achieving dynamic color tuning [29,30].

In this study, we use Sn as a catalyst to synthesize Sn-doped CdS nanowires. By controlling the Sn-doping concentration and growth environment, Sn-doped CdS nanowires with different Sn-doped concentrations can be obtained. We analyzed the effect of Sn-doped concentration on the excitation-power-dependent color-tuning process. Above all, it was found that the multicolor dynamic control is realized in Sn-doped CdS nanowires with weight ratios of 10:1 of CdS and SnO_2_. The color reflects red, orange, yellow and green, in turn, in individual Sn-doped CdS nanowires, which results in an increase in excitation power. Its photoluminescence (PL) spectrum shows a broad trap-state emission band out of Sn dopants, which is superposed by a microcavity due to the nanowires’ size and structure, besides the CdS band-edge emission. As the number of light-emitting peaks increases, the intermediate colors of orange and yellow are easily displayed. Therefore, this Sn-doped CdS nanowire will provide an effective solution to increase the color spectral range of color-tuning devices that depend on excitation power.

## 2. Results and Discussion

### 2.1. Morphology

**Figure 1:**
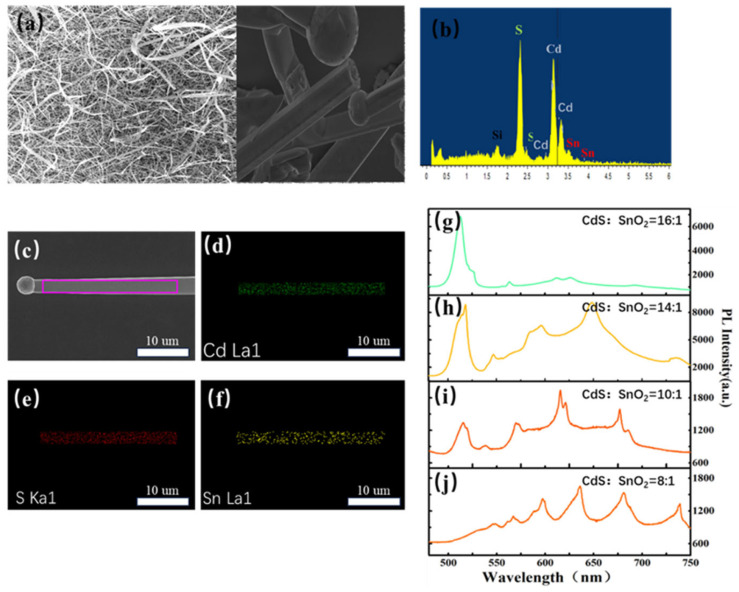
Morphology of CdS nanowire (**a**). SEM image of Sn-doped CdS nanowire dispersed on Si substrate. (**b**) EDS profiles of a typical Sn-doped CdS nanowire in (**a**). (**c**) The high magnification image of Sn-doped CdS nanowire. (**d**–**f**) The SEM elements mapping Cd, S and Sn. (**g**–**j**) PL spectra of samples with different ratio of CdS and SnO_2_.

Figure 1a is the SEM morphology of Sn-doped CdS nanowires on a transparent SiO_2_ wafer. The nanowire has smooth surfaces and lengths up to tens of micrometers. In addition, some nanowires have big spheres on the top, revealing their vapor–liquid–solid (VLS) growth mechanism. Furthermore, EDS profiles of the Sn-doped CdS nanowire are demonstrated in Figure 1b, which indicates the element species of the Sn-doped CdS nanowires. The Sn-doped CdS nanowire consists of of three elements, namely Cd, S and Sn. X-ray mappings of the area in the purple box in Figure 1d–f show Cd, S and Sn are uniformly distributed in the Sn-doped CdS nanowire. Figure 1g–j shows the PL spectra of samples A, B, C and D. The PL spectra showed that all the samples have band-edge emission bands at 520 nm and broad emission bands spanning from 540 nm to 750 nm. Wide emission originates from trap state emission caused by Sn doping. Due to different Sn concentrations, the ratio of the emission intensity of trap-state emission to band-edge emission increases significantly from sample A to sample D.

### 2.2. The Structure of the Sn-Doped CdS Nanowire

**Figure 2:**
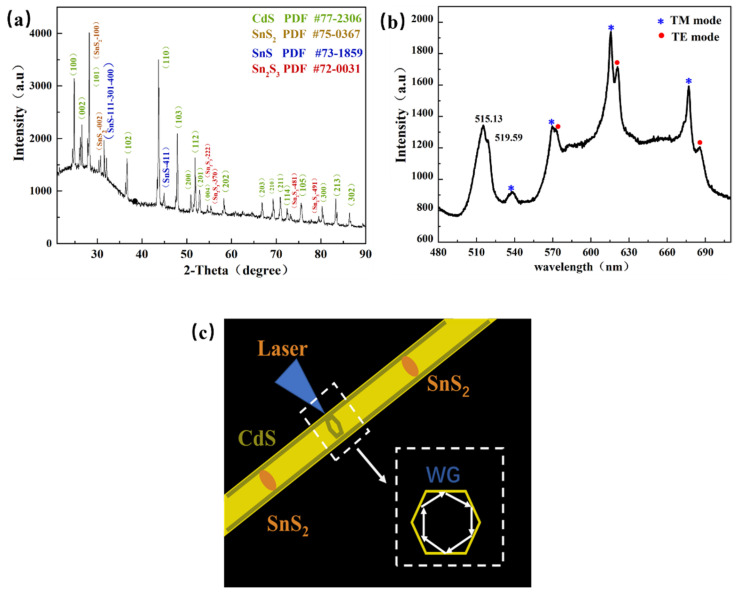
The structure of the Sn-doped CdS nanowire. (**a**) XRD spectrum of Sn-doped CdS nanowire. (**b**) PL spectrum of the Sn-doped CdS nanowire at the excitation power of 0.251 mW. (**c**) Schematic illustration of the luminescence of Sn-doped CdS nanostructure at room temperature.

Although trap-state emission and band-edge emission were observed in these Sn-doped CdS nanowires, especially for sample in Figure 1j, with almost all the emission coming from the trap-state emission, the dopant contents (Sn element) and the existence of Sn dopants are still too different to be detected by the EDS with a sensitivity of 1%. Here, we use XRD spectra from individual nanowires to confirm the existence of Sn dopants. Meanwhile, we use the PL spectrum to confirm the structure of the nanowire. Figure 2a is the XRD spectrum of the Sn-doped CdS nanowire, and it indicates that the nanowire is a wurtzite structure. All peaks match with the Joint Committee on Powder Diffraction Standards (JCPDS) card No. 75-0367, No. 73-1859 and No. 72-0031. Thus, the XRD spectrum from individual Sn-doped CdS nanowires shows the valence states of Sn dopants. Three main states, namely SnS_2_, SnS and Sn_2_S_3_, were founded. Figure 2b shows the PL spectrum of a typical Sn-doped CdS nanowire with an excitation power of 0.251 mW at room temperature. The band-edge emissions have two fitted peaks at 515.13 nm and 519.59 nm. The emission of 515.13 nm is the band-edge emission of CdS. The emission of 519.59 nm comes from a new bound exciton, possibly due to the SnS_2_, Sn_2_S_3_ and SnS species inside the nanowire [31]. In the trap-state emission from 540 nm to 750 nm, the PL spectrum shows a series of sharp emission peaks and weak emission peaks. These peaks might be attributed to microcavities that come from the resonances of the trap-state lighting around the side walls (shell layers) of the Sn-doped CdS nanowire [32]. Generally, there are two types of microcavities, namely Fabry–Perot (F–P) microcavity and whispering-gallery (WG) microcavity. Because the PL spectra were collected in situ, the PL spectrum signal emitted in the longitudinal direction is difficult to collect. These emission peaks originate from WG microcavities rather than F–P microcavities (Figure 2c). A plane wave model of WGM was used to investigate the polarization mode of these observed WG microcavities. The mode numbers of TE-WGM (the electrical component of light E⊥c-axis) and TM-WGM (the electrical component of light E∥c-axis) can be calculated by the following equations [33]:

(1) $$\lambda_{TM-WGM}=\frac{3\sqrt{3}nR}{N+\frac{6}{\pi }tan^{-1}\left(\frac{1}{n}\sqrt{3n^{2}-4}\right)}$$

(2) $$\lambda_{TE-WGM}=\frac{3\sqrt{3}nR}{\left(N-3\right)+\frac{6}{\pi }tan^{-1}\left(n\sqrt{3n^{2}-4}\right)}$$

where *n* is the mode number, *R* is the side length of a hexagonal microcavity and *R* is 1.03 μm in this paper; *n* is the refractive index. Due to the negligible doping of SnO_2_, the refractive index *n* (E) of CdS was used in the calculation, which is 2.13 [34]. According to the PL spectrum, the wavelength-dependent mode numbers were separately calculated for different polarization modes (*TM* and *TE*) of the nanowire, the calculated *TE* mode number changed from 11 to 14 and the *TM* mode number changed from 10 to 13 [35]. The sharp modes (see the peaks marked with stars in Figure 2b) are consistent with the calculated *TM* modes, while the weak modes (see the peaks marked with circles in Figure 2b) are consistent with the calculated *TE* modes [36]. These are good agreements between the calculation and the PL spectrum.

### 2.3. Optical Lighting Behavior Sn-Doped CdS Nanowires with the Increase of Sn-Doping Concentration

**Figure 3:**
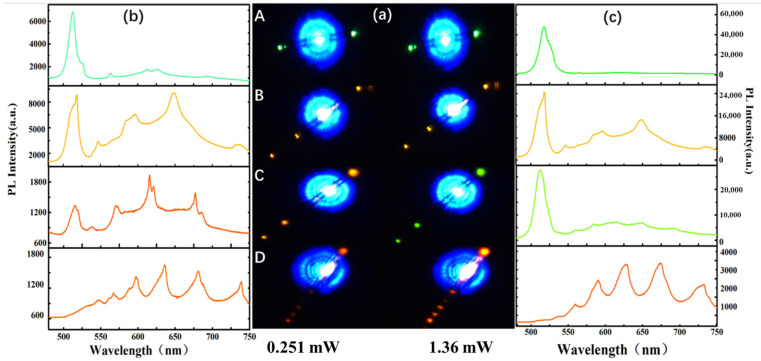
Optical lighting behavior of Sn-doped CdS nanowires with different CdS and SnO_2_ weight ratios. (**a**) Real-color PL image with focused excitation (405 nm) of each Sn-doped CdS nanowires, left is 0.576 mW, right is 1.36 mW. (**b**,**c**) PL spectra recorded at 0.251 mW and 1.36 mW, as followed by samples A, B, C and D, respectively.

To study the excitation power-dependent color-tuning behavior related to doping concentration, we compared the optical lighting behavior of Sn-doped CdS nanowires with four different Sn-doping concentrations, namely samples A, B, C and D. Figure 3a shows the real-color PL images of the four samples with the excitation power of 0.251 mW and 1.36 mW. Figure 3b,c show the PL spectra of the four samples at the excitation power of 0.251 mW and 1.36 mW, respectively. It can be seen that the band-edge emissions are decreasing, and the trap-state emissions are increasing with the increases in Sn-doping concentration. Therefore, the higher the Sn concentration in Sn-doped CdS nanowires, the easier it is to display red lighting. For sample A, the band-edge emissions are both dominant with the excitation power of 0.251 mW and 1.36 mW, so the colors are green. For sample B, the ratio of the emission intensity of trap-state emission and band-edge emission with the excitation power of 1.36 mW is slightly reduced compared with the ratio of the emission intensity of trap-state emission and band-edge emission with the excitation power of 0.251 mW. Because red chroma is stronger than green, the color we see to the naked eye is red. For sample C, the trap-state emission dominates at 0.576 mW, but the band-edge emission dominates at 1.36 mW. The ratio of the emission intensity of trap-state emission to band-edge emission has changed greatly, and we can see the emission colors are red with an excitation power of 0.251 mW and green with an excitation power of 1.36 mW. For sample D, the trap-state emissions are both dominant with an excitation power of 0.251 mW and 1.36 mW, so the colors are green. Therefore, it is of great significance to find the appropriate Sn-doping concentration for the excitation power-dependent color tuning. Sn-doped CdS nanowire with CdS and SnO_2_ weight ratios of 10:1 in this paper can obtain color tuning between 0.251 mW and 1.36 mW.

### 2.4. Real-Color Image of Excitation-Power-Dependent Color Tuning and Corresponding PL Spectra of the Sn-Doped CdS Nanowire

**Figure 4:**
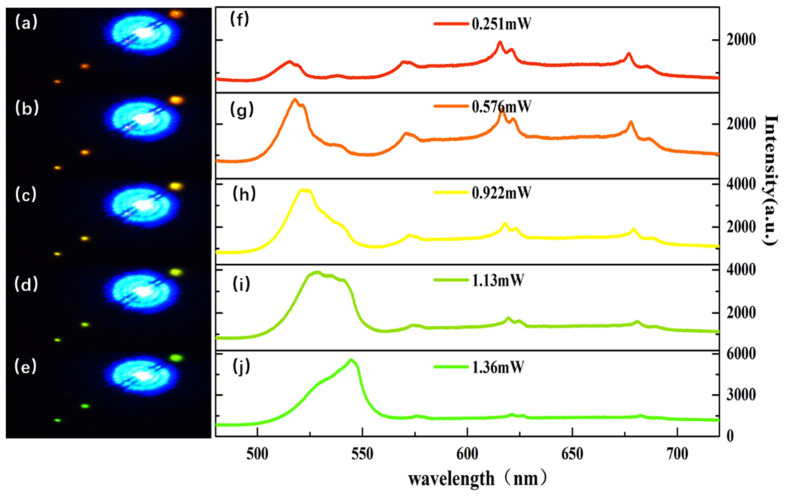
Real-color image of excitation-power-dependent color tuning and corresponding PL spectra of the Sn-doped CdS nanowire (**a**–**e**). The real-color image at 0.251 mW, 0.576 mW, 0.922 mW, 1.13 mW and 1.36 mW. (**f**–**j**) The corresponding PL spectra.

To understand the principle of color-tuning emission, the excitation power-dependent color-tuning process of Sn-doped CdS nanowires with a CdS and SnO_2_ weight ratio of 10:1 was measured in detail. Figure 4a–e show the real-color images of the Sn-doped CdS nanowire, which is under the different excitation-power intensities of 0.251 mW, 0.576 mW, 0.922 mW, 1.13 mW and 1.36 mW, respectively. Figure 4f–j show the corresponding PL spectra at five different excitation power intensities. All the PL spectrum shows resolvable luminescence from four sharp emission peaks, which are centered at 520 nm (green emission), 570 nm (yellow emission), 615 nm (orange emission) and 670 nm (red emission), respectively. When Sn-doped CdS nanowire is stimulated by excitation power is 0.251 mW. The PL intensity of the yellow emission, orange emission and red emission are larger than that of the green emissions. Because red chroma is stronger than others, the corresponding emission image exhibits a red color, as shown in Figure 4a. With the excitation power intensity increasing to 0.576 mW and 0.922 mW, the difference in PL intensity between the four-color emission becomes smaller, as shown in Figure 4g,h. The emission colors are represented as the intermediate colors between green and red, which are close to orange in Figure 4b and yellow in Figure 4c. When the excitation power intensity reaches 1.13 mW and 1.36 mW, the PL intensity of the green emission is dominant, as shown in Figure 4i,j, which shows a clearly green-color image. Therefore, the Sn-doped CdS nanowire could tune the emission color from red to orange to yellow to green with the increase of excitation power. Different from the color-tuning effect of In-doped CdS nanowires and Sn-doped CdS comb-like nanostructure, the tunable color of the Sn-doped CdS nanowire is more precious.

### 2.5. The Mechanism of Lighting Emission at Different Wavelengths

**Figure 5:**
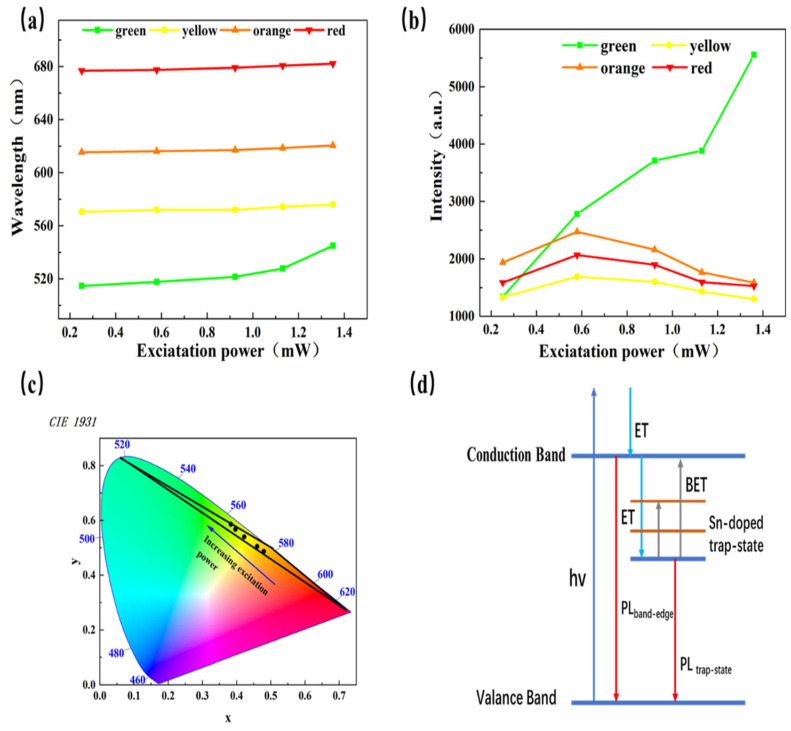
The mechanism of lighting emission. (**a**) Excitation-power-dependent wavelength shift of four-color emission peak. (**b**) Excitation-power-dependent intensity shift of four-color emission peak. (**c**) The CIE chromaticity diagram of Sn-doped CdS nanowire. (**d**) Excitation-power-dependent energy level of the transition radiation process of Sn-doped CdS nanowire.

To understand the mechanism of lighting emission at different wavelengths and the transition radiation process of Sn-doped CdS nanowire, we extracted the two light-emitting background, namely, the excitation-power-dependent wavelength shift in Figure 5a and excitation-power-dependent intensity shift in Figure 5b. By analyzing the background, the structure information and the interactions information between exciton–carrier and exciton–phonon could be obtained. In Figure 5a, the wavelengths of green emission, yellow emission, orange emission and red emission red-shift from 514.6 nm to 544.9 nm, 570.4 nm to 575.9 nm, 615.5 nm to 620.5 nm and 676.8 nm to 682.2 nm, respectively, and corresponding the slope are about 7.059, 1.331, 1.240 and 1.386, respectively. With the increase of excitation power, the green emission rate increases faster compared to others. As the green emission comes from the band-edge emission, more free excitons can be generated in Sn-doped CdS nanowires with the increase of excitation power. Some of the free excitons are transformed into free electrons and holes at higher powers by scattering between carriers, resulting in enhanced interaction between electrons and phonons and expansion of the crystal lattice. These processes will cause the conduction band and valence band to move, which makes the band-edge emission red-shift significantly. This red-shifted characteristic with band-edge emission is consistent with the characteristic among most II–VI semiconductor nanostructures, such as In-doped ZnTe microstructures and ZnO nanowires [37,38]. The drift slopes of the red emission, orange emission and yellow emission are approximately equal to 1; that is, there is no obvious red-shift phenomenon with the increase of excitation power. Previous studies found that the power change has little effect on trap-state emissions and microcavity emissions. Therefore, the emissions of red, orange and yellow should come from the superposition of microcavities and defect states, which is consistent with the PL spectrum analysis.

Figure 5b shows that the green emission intensity shifted from 1130 at 0.251 mW to 5550 at 1.36 mW. However, the other emission intensity rises first and then falls. When the excitation power is 0.576 mW, the intensity is maximum. The peak intensity emissions of yellow, orange and red are 1690, 2471 and 2067, respectively. Meanwhile, we note that the strongest mode shifts from 615.5 nm with an excitation power of 0.251 mW to 540 nm with an excitation power of 1.36 mW. When the excitation power is low (0.251 mW), there are three main reasons why the intensities of the red, orange and yellow emission dominate compared with the green emission intensity. Firstly, the coupling of the trap-state emission and the microcavity mode significantly enhances the intensity of the mixed emission, and this microcavity mode does not exist in the previously reported Sn-doped CdS multi-branched nanowire. Secondly, Sn-ions can form a plasma with carriers at lower power, leading to burst emission of trap states and microcavities. Finally, at lower excitation power, the low carrier concentration reduces carrier scattering and quenching, which effectively amplifies the microcavity mode emission intensity. With the increase of excitation power, the electron hole plasma can firmly couple carriers and can generate more free excitons, resulting in high energy in the green emission. Therefore, when the excitation power is high, the green emission can obtain high gain. However, the intensity of red, orange, and yellow emission lighting is reduced due to microcavity loss. Therefore, the green emission intensity increases with the increase of excitation power, while the red, orange and yellow emission intensities first increase and then fall with the increase of excitation power. In Figure 5c, the chromaticity of the spectra in Figure 4a–e are marked on the CIE chromaticity diagram. The colors calculated in Figure 5c exactly match the colors in the real-color images shown in Figure 4a–e, further verifying the monochromaticity and color tuning of the Sn-doped CdS nanowires. The area of the black triangle box in the CIE chromaticity diagram is the range of the spectrum of dynamic color tuning, which greatly expands the color spectrum compared to the spectrum of traditional color-tuning nanostructures. In addition, any color on this black triangle box can be obtained from the Sn-doped CdS nanowires by precisely tuning the excitation power.

Based on the above relationship between the excitation-power-dependent wavelength shift and excitation-power-dependent intensity shift, we summarized the transition radiation process of Sn-doped CdS nanowires with the increase of excitation power. At a low excitation power, electrons are excited from the valance band (VB) to the conduction band (CB), and the excited electrons relax to the bottom of the conduction band (CBM) through the energy transfer process. Some of the electrons recombine with holes in the valance band, while others relax to the trap-state level introduced by Sn doping and then recombine with holes in the valance band. At high excitation power, the Sn-doped CdS nanowires can generate more phonons, resulting in a thermal effect. With the thermal equilibrium in the lattice of Sn-doped CdS nanowires, a back-energy transfer (BET) process may occur between the trap-state level introduced by Sn-doping and the shallow bound energy level near the conduction band [39,40]. According to the BET process, we can also explain why the intensity of the red emission increases first and then decreases with the increase of excitation power. At relatively low excitation power, because fewer electrons are captured by the shallow bound state near the conduction band and generating fewer phonons, the probability of the BET process occurring is smaller. At this time, the intensity of the green emission and the red emission are both increasing with the increase of excitation power. When the excitation power increases to a higher value, more phonons are generated, and the probability of the BET process occurring is larger. Most electrons are excited into the conduction band, resulting in the intensity of the green emission increasing rapidly and the intensity of the yellow emission, orange emission and red emission decreasing. At the same time, the rest of the electrons captured by the trap-state level may return to the shallow bound state near the conduction band, resulting in an increase in green emission intensity and wavelength red-shifting with the increase of excitation power.

## 3. Experimental Section

### 3.1. Fabrication of the Sn-Doped CdS Nanowires

The Sn-doped CdS nanowires were synthesized using a simple chemical vapor deposition (CVD) method with Sn as a catalyst. First, the mixtures of CdS and SnO_2_ were put into a ceramic boat, and the ceramic boat was placed into the central area of the quartz tube. The mixtures of CdS and SnO_2_ weight ratios are 16:1,14:1,10:1 and 8:1. These related products are marked as samples A, B, C and D, respectively. In other words, the Sn concentration in Sn-doped CdS nanowires increases from sample A to sample D. First, mica pieces ultrasonically cleaned in acetone, ethanol and deionized water were placed downstream of the quartz tube, about 11 cm away from the central area of the quartz tube. Second, the quartz tube was pulled into the heating zone of the tube furnace. The high-purity mixed gas with 90% argon and 10% hydrogen flowed through the quartz tube at a flow rate of 60 sccm for one hour to purify the growth environment of the nanowires. Third, the tube furnace temperature was heated to 850 °C at a rate of 100 °C/min and held at that temperature for 40 min with a constant gas flow of 30 sccm and an atmospheric pressure of 300 mbar. When the work finished, and the tube furnace temperature naturally dropped to room temperature, yellow-colored Sn-doped CdS nanowires were deposited on the mica pieces. Finally, the Sn-doped CdS nanowires were dispersed on a clean SiO_2_ wafer.

### 3.2. Characterization

After the synthesis, the morphology and microstructural information of the Sn-doped CdS nanowires were characterized by scanning electron microscopy (SEM, Zeiss SUPRA 55, Jena, Germany) and energy dispersive X-ray spectroscopy (EDS), respectively. The valence states of the elements and the chemical stoichiometry of the Sn-doped CdS nanowires were both characterized by X-ray diffraction spectroscopy (XRD, JEOL JPS-9010MC, Akishima, Japan). Photoluminescence (PL) spectra with the increase of excitation power were characterized by a confocal micro-PL system (Princeton Instruments Acton SP2500, Acton, MA, USA, Olympus BX51M, Tokyo, Japan) with a continuous-wave (CW) GaN laser 405 nm as the excitation source. The in situ real-color images were recorded using a charge-coupled device (CCD) camera (Beijing, China).

## 4. Conclusions

In summary, the high-quality Sn-doped CdS nanowires are successfully synthesized by a simple chemical vapor deposition method. These unique Sn-doped CdS nanowires with the weight ratios of CdS and SnO_2_ RW 10:1, we have further demonstrated that four-color emission can be achieved with the increase of excitation power the first time. The real-color images show that the emission color turns from red, orange, and yellow to green by increasing the excitation power. The dynamics behavior of excitation-power-dependent wavelength shift demonstrates that the green emission originates from the band-edge exciton recombination, and the red, orange and yellow emissions are related to the trap-state recombination and the formation of microcavity. More importantly, suitable Sn-doping concentration and the introduction of microcavity in nanowires allows for dynamic control or tuning of the combined colors in a precious range. We believe that Sn-doped CdS nanowires with four tuning colors will provide many opportunities for applications such as lighting, displays, light emission and frequency conversion equipment.

## Data Availability

Data are contained within the article.
